# The Cost of a Medical Education

**Published:** 1893-09-16

**Authors:** 


### The Cost of a Medical Education.

Parents and guardians in selecting the particular walk of life to be followed by the young men whose welfare is concerned, must be guided by three considerations. Is the profession or occupation suitable as regards position and the means of living it will provide? Is my son suitable for this or that profession? Can I afford the necessary expense in educating and starting him in the profession?

Without attempting to go into detail, we may be able to answer some of the questions that naturally present themselves to those who desire to make their sons practitioners of medicine.

It is not a difficult matter to become a qualified medical man, for any young man of average capacity and ordinary application will pass the necessary examinations. But there is a very great difference between the status of the various qualifications and qualifying bodies, and a corresponding difference between the standard of the examinations.

The first thing is to pass the preliminary examination in arts. Here, at the outset, the student must decide whether he will be content with the lowest qualifications, or will aspire to the higher and more difficult diplomas and degrees. Thus, for the London University degrees, he must matriculate at the University, or pass an equivalent examination (those which are accepted as equivalent may be ascertained by reference to the University calendar), whereas for the double qualification of the Royal Colleges of Physicians and Surgeons, a far lower arts examination is accepted. In former days, when Oxford and Cambridge were nearly the only universities which granted medical degrees, the expense was great. Residence for several years was required, and a degree in arts necessary before the student could proceed to the medical degrees. Only at Oxford is this latter now required, and several universities require little or no residence within a college connected with it.

The Universities of Durham, Glasgow, Aberdeen, are less exacting in their preliminary arts, and even Edinburgh is certainly far less difficult in its university matriculation than is the London University.

It is well, therefore, to recognize at the outset that if the intending student has only an average mental capacity, the higher examinations of the London University and the Fellowship of the College of Surgeons are beyond his powers, and that it is only waste of time and money to try for that which is beyond his range. On the other hand, it is a good thing to remember that no man knows what he can do till he tries, and it is well to encourage a student to aim high, and be filled with a worthy ambition to excel.

What is the cost of a medical education? The minimum time in which a student can become a qualified practitioner is five years, and therefore the parent must reckon on the cost of keeping him for five years. What this will come to it is quite impossible to say, for the simple reason that the requirements of individuals vary so greatly that what one student would "starve" on another would consider an ample allowance. It is possible to get a single room in London, conveniently situated, for about ten shillings a week, or two rooms at fifteen shillings. One cannot live under a pound a week at least for food, coals, and gas. Thus a very economical student who could "rough" it, can live for about £80 a year, for board and lodging. But the majority of students could not exist comfortably on less than £100 a year, amounting to five hundred in five years. The amount required for clothes and petty expenses can be calculated by each parent.

But the total amount of fees is very much easier to determine exactly, although these vary considerably. The composition fee, which includes the fees for all lectures and hospital work, varies from 110 guineas to 150 guineas at the different metropolitan schools, while at the provincial schools the composition fee is rather lower; thus at the Bristol Medical School it is only £94 10s. Additional expenses, such as cost of parts in anatomy, instruments, books, subscriptions, additional lectures, &c., amount to about £30. The double qualification diploma fees amount to £36 10s., and additional qualifications about £22 each extra. Thus the total cost of education and necessary expenses, exclusive of board and lodging, amount to about £140 to £200.

We may say that since, as a rule, the wealthier students go to the more expensive schools, the other expenses will be roughly proportional to the entrance fees, for it is very difficult for a student to live differently from the fellow students, for if he succeeds he generally does so at the risk of losing all the advantages of warm friendships and pleasant intercourse, which constitute one of the most important factors in the early training of young men.

The question whether a student should enter at a provincial or a metropolitan school is one which must exercise the mind of every parent or guardian who wishes to give his son an English diploma or degree.

The question hardly applies to Scotland or Ireland, for the schools there educate almost entirely for the particular examination with which the college is connected. There are now several excellent medical schools scattered about England: Bristol, Birmingham, Bradford, Leeds, Manchester, and Newcastle; all supplied with energetic and capable teachers, and equipped with all the most modern appliances for teaching the numerous branches of medical study. The parent may well ask himself whether he is not doing his best for his son in sending him to one of these schools rather than to a London one. The main question is one of expense. There is little doubt that the cost of keeping a young man in the provinces is less than it is in London, to say nothing of the opportunities there are of keeping an eye on his conduct and care, and helping him to avoid the many temptations that come in the way of a young man for the first time feeling his liberty. But it must be admitted that the provincial schools have as yet not taken that hold of the general public or of the medical profession which their course of study and general efficiency warrants. However good a man may be at his work, it is of great value to him to have the power to say he was at a London medical school. He may be as well taught, have had greater facilities for clinical study and practical work, but the fact that he was "only" a provincial student will weigh against him. We may at once say we have the highest opinion of the provincial schools and their teaching, but it is an undoubted fact that they have not the same status as the London schools. In the selection of a hospital the question of fees may generally be put aside, for the difference between the charges of the various London schools is very small. What should be aimed at in selection is to choose one which affords the greatest facilities for clinical study. It may sound contradictory, but this requisite is not always to be found at the hospital which contains the greatest number of beds. It will be observed that at the largest hospital are the greatest number of students, and as it is essential in clinical work to be able to examine a patient quietly, it often happens that the greater the number of patients the less chance there is of doing this. Besides this, the large number of patients seen at the visit of a physician or surgeon to the wards makes it almost an impossibility for the student to follow the course of the various diseases brought under his notice. At a small school he will, by diligent attendance in the wards, see all the commoner diseases in the course of his clinical work, and further, he will be able to follow the course of the cases and of the treatment, and remember the changes in the patient's condition far better than if he attempts to crowd his brain with a superfluity of cases. He will farther have the opportunity of getting much more personal supervision from the members of the staff, many of whom he may become intimate with. The student will find ample time during his course to attend the hospitals for special diseases, and this he is strongly recommended to do. The general hospitals have, to a considerable extent, in the past neglected their special departments, and in consequence a number of eye, throat, and other hospitals have been founded. Students can always attend the out-patient department of these institutions; and at many of them regular courses of lectures and demonstrations are held.

